# Anti-Hypochlorite and Catalytic Activity of Commercially Available *Moringa oleifera* Diet Supplement

**DOI:** 10.3390/molecules24183330

**Published:** 2019-09-12

**Authors:** Karolina Starzak, Bernadette Creaven, Arkadiusz Matwijczuk, Alicja Matwijczuk, Dariusz Karcz

**Affiliations:** 1 Department of Analytical Chemistry (C1), Faculty of Chemical Engineering and Technology, Cracow University of Technology, Warszawska 24, 31-155 Cracow, Poland; dkarcz@chemia.pk.edu.pl; 2 Centre of Applied Science for Health, Technological University Tallaght, Dublin 24, Ireland; Bernie.Creaven@tudublin.ie; 3 Department of Biophysics, University of Life Sciences in Lublin, Akademicka 13, 20-950 Lublin, Poland; arkadiusz.matwijczuk@up.lublin.pl (A.M.); alicja.matwijczuk@up.lublin.pl (A.M.)

**Keywords:** *Moringa oleifera*, hypochlorite, scavenging, free radicals, antioxidative potential, inflammation, coumarins, DPPH

## Abstract

Aiming at the assessment of the pro-health, and especially anti-hypochlorite properties of Moringa oleifera species a representative, commercially available *Moringa oleifera* dietary supplement was used as a substrate for the preparation of aqueous Moringa extract. The anti-hypochlorite activity of the extract was assessed using the hypochlorite-specific coumarin-based fluorescence turn-off sensor, namely 7-diethylamino-coumarin-3-carboxylic acid (7-DCCA). This compound was synthesized via the Knoevenagel condensation of 4-diethylamino-2-hydroxybenzaldehyde with Meldrum’s acid and the Moringa extract was employed as a medium and catalyst. Moreover, the total phenolic content (TPC) as well as the reactive oxygen species (ROS)–scavenging ability of the aqueous Moringa extract were determined. The results obtained demonstrated the applicability of Moringa extract as an anti-hypochlorite agent. Additionally, the satisfactory yield of the 7-DCCA obtained suggests the usefulness of the extract as a catalyst and the reaction medium. The antioxidative potential of the extract was notably lower than that of the standard (TROLOX). Determination of TPC in 100 g of the dry weight (DW) of studied material revealed a high number of polyphones present.

## 1. Introduction

Moringa (*Moringa oleifera*), commonly known as a ‘drumstick tree’ or ‘horseradish tree,’ is a tree belonging to the species from the Moringa family (*Moringaceae*) and endemic to subtropical and tropical regions of the world. It is often called the “tree of life” because of a high level of various nutrients in its leaves, pods, and seeds [1]. The ease with which it undergoes cultivation makes it an extremely valuable source of nutritional ingredients, especially in regions of the world threatened by malnutrition [2]. In developing countries, various parts of the Moringa tree are exploited with the leaves being the most commonly used [3]. The multitude of applications associated with Moringa includes water and oil purification, biomass for biodiesel production, animal feeding, fencing, local medicines, and the firewood [2,3]. Moringa leaves are particularly rich in vitamins, antioxidants such as flavonoids, carotenoids, ascorbic acid, polyphenols, as well as tannins, saponins, proteins, and minerals. Hence, they are commonly used in food preparations. Asian and African local medicine uses them for treating various conditions such as malaria, parasitic diseases, typhoid fever, swelling, skin problems, arthritis, hypertension, strengthening the immune system, eliciting lactation, and many others [2,3,4]. High level of zeatin in Moringa leaves makes the leaves’ extract a natural plant growth enhancer [2]. Other parts of the Moringa tree have been reported as lowering cholesterol levels in humans, and showing diuretic, antispasmodic, antiulcer, antitumor, anticancer, antibacterial, antifungal, and hepatoprotective activities [4].

In order to profit from beneficial properties of Moringa, markets around the world are full of various medicinal, dietetic, and cosmetic preparations, available for people living in areas where Moringa cultivation is impossible. Most of these diet supplements are capsules containing simple powdered Moringa leaves. In our studies, we examined one of diet supplements from Cyprus available on the market in central Europe in order to establish its antioxidant capacity and determine total phenolic content in a commercially available product.

Our main goal was to check whether such products exhibit at least rudimentary activity attributed to Moringa. Second, several research reports point at Moringa as exhibiting anti-inflammatory and immunomodulatory effects [2], yet none of them focused on OCl^−^ (inflammation marker) elimination by leaves extract. In addition, aiming at the examination of catalytic potential of the Moringa extract a modified Knoevenagel condensation methodology was applied for the synthesis of 7-DCCA. Specifically, a green chemical approach for the synthesis of coumarin carboxylic acids [5] inspired us to use an aqueous Moringa extract as a medium and catalyst for the condensation of 4-diethylamino-2-hydroxybenzaldehyde with Meldrum’s acid.

Taking into account, that most Moringa-based supplements available in Europe consist solely of powdered Moringa leaves, the food supplement employed as a substrate in our studies was considered representative. On the other hand, it is noteworthy that depending on the geographical origin or cultivation conditions of the plant, the results obtained may exhibit slight differences [6,7,8].

## 2. Results and Discussion

### 2.1. Total Phenolic Content Determination

Polyphenols are considered to be responsible for the natural, antioxidant properties of plant products. Hence, an investigation of their content in diet supplements seems necessary. Various research on total phenolic content (TPC) in *Moringa oleifera* leaves show that their final content depends on the variety of the plant, the season in which the plant material was harvested, and the location and, therefore, climatic conditions at which it grew [6,7,8]. However, most of the available research data were obtained with various organic solvents used for polyphenols extraction.

A modified Follin–Ciocalteu method, using gallic acid as a standard reference compound and results expressed as gallic acid equivalents (GAE), was applied to determine TPC of Moringa leaves extract. The amount of polyphenols determined in 100 g of the dry weight (DW) of studied material was 4.04 ± 0.11 g GAE. The value obtained is lower compared to that of methanolic extracts 9.95–11.17 g/100 g DW [6] and similar to those of acetone extracts 2.87–3.19 g/100 g DW [7]. Aiming at obtaining the relevant results in terms of aqueous conditions prevailing in the human body, all of our experiments were performed using water as an extracting agent. This resulted in lower polyphenol content determined most likely due to the poor aqueous solubility of various organic compounds, present in Moringa leaves [2,9]. On the other hand, the comparison of the TPC value obtained with those reported for various seasonings, fruits, vegetables, or seeds clearly state that TPC in Moringa is one of the highest of all plants tested [7,8,10,11]. Among more than 100 plant products tested only cloves, dried peppermint, star anise [11], and European elder [10] exhibited the TPC value higher than that of Moringa. This implies that, regardless of the sparing aqueous solubility of polyphenols, the high TPC content in Moringa leaves allows for transfer of these compounds into aqueous solution at levels beneficial to human health. Moreover, even a diet supplement available commercially in the area where Moringa species do not occur naturally contains a satisfactory amount of polyphenols.

### 2.2. Antioxidative Assay

The physiological conditions inside the human body are diverse and span from strongly acidic elements in the initial parts of the digestive system (pH 2–3), through weak acidic conditions occurring inside the white blood cells (pH 5.0–5.5) [12,13], to slightly alkaline pH of body fluids (pH 7.4). Taking into account the fact that the dietary supplements are usually administered orally, and their active ingredients are transported with blood and physiological fluids, they must have the ability to withstand the changing pH conditions. All further analyses were performed at three various pH conditions, which include 3, 5, and 7.4.

Determination of antioxidative potential was performed with a standard spectrophotometric test using DPPH^•^ radicals solution. The rate at which the solutions of analytes tested reduce the constant concentration of radicals determines the antioxidant capacity of the compounds tested. The results obtained are compared to the standard that is the ethanolic TROLOX solution. The DPPH^•^ radical reduction method was utilized due to the fact that, in the wavelength range in which the measurements are performed, Moringa extracts do not exhibit any absorption bands.

**Figure 1:**
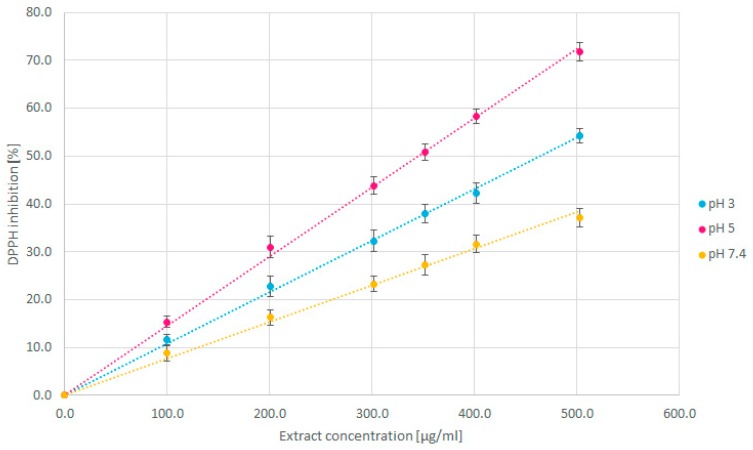
DPPH^•^ reduction percentage of aqueous Moringa extracts at various pH values after 30 minutes of reaction at 25 °C.

Figure 1 shows the percentage of DPPH^•^ reduced at various pH values in the presence of increasing concentrations of Moringa extract. The slope indicates the antioxidant capacity in an inverse relationship between the percentage of DPPH^•^ inhibited and concentration of the Moringa extract. In more detail, the lower the extract concentration required to reduce a fixed number of radicals, the higher the antioxidant capacity of the extract tested. Similar graphs for ascorbic acid and TROLOX are included in Supplementary files (Figures S1 and S2, respectively).

Previous studies proved that changes in H^+^ concentration lead to changes in the mechanism of DPPH scavenging by polyphenols. Increase in the pH value (in acidic range) leads to a decrease of the reaction rate between extract components and DPPH radicals [14,15]. Thus, higher DPPH inhibition observed at a pH of 5 than at a pH of 3 (Figure 1) results from lower antioxidant activity of the Moringa extract observed in less acidic media.

**Figure 2:**
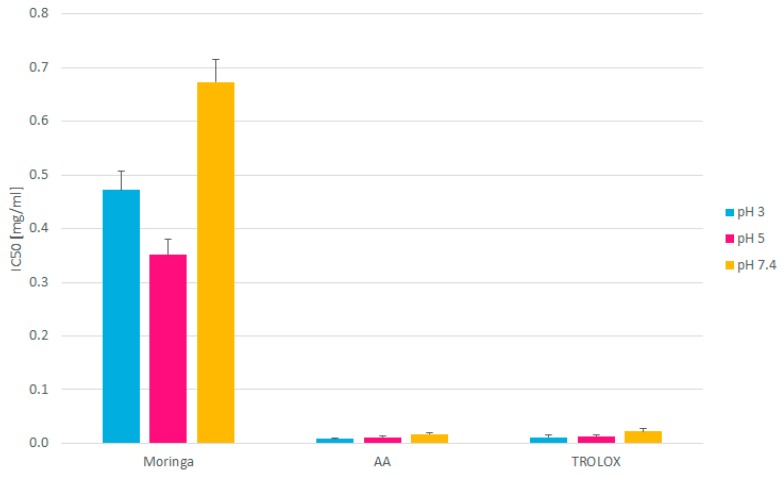
IC_50_ values of Moringa extracts, ascorbic acid (AA), and TROLOX at various pH values.

Based on the results obtained, the IC_50_ value determining the analyte concentration required to reduce fixed, initial radical concentration by 50%, was calculated (Figure 2).

The IC_50_ values for Moringa leaves extracts at each pH tested were significantly higher, compared to those of pure ascorbic acid (AA) and TROLOX, which indicates their much lower antioxidant activity. The relatively low values may also be a consequence of water being used as an extracting solvent for raw, plant material. It is well-known that the antioxidant properties of plant products originate mainly from polyphenols and flavonoids, which are poorly (if at all) soluble in water. The existing studies show that organic solvents used for the extraction increase the final yield of polyphenols extracted, which increases the antioxidant capacity of extracts tested [16]. Investigations into Moringa extracts obtained using various solvents state that the extraction with moderate polarity solvents, such as butanol or ethanol gives the best results in terms of antioxidant properties of the final extract [17]. However, the use of such solvents to prepare a product for use in a health shop or as a food supplement would be limited. While typically pharmaceutical medication is taken in milligrams, food supplements and health food capsules tend to be taken in grams. Therefore, the relative comparison of antioxidant activity is difficult to make. It is also very likely that, in our studies, the compounds with the ability to reduce DPPH^•^ were not fully extracted from plant material, and, hence, the aqueous Moringa extracts obtained demonstrated the relatively poor DPPH^•^-scavenging ability. On the other hand, it is worth emphasizing that the reason for using aqueous extract in our research was mimicking in vitro physiological conditions, which prevail in humans, especially in those body parts that interact with food supplements. In this context, the previously reported IC_50_ value for aqueous Moringa extract (0.517 mg/mL) [17] correlates well with those obtained from our studies (0.472 mg/mL, 0.351 mg/mL, and 0.673 mg/mL at the pH of 3, 5, and 7.4, respectively).

Another, although highly unlikely, reason for low antioxidant activity of the Moringa extract tested is the quality of the substrate. Based on the description of the product provided by the manufacturer on the packaging, one can assume that the substrate consists solely of powdered *Moringa oleifera* leaves and no additional supplementary substances are added. Information on where the plant material comes from, at what stage of vegetative development the plant has been harvested [2,6,18], and how it is stored, can have a significant impact on the final results as well. The IC_50_ value of aqueous extract similar to that of Reference [17] implies that the substrate used in our studies does not contain any additional substances or the additives present (if any) did not affect the IC_50_ value. This hypothesis, however, remains unverified, due to the lack of information regarding the nature of potential additives present in the food supplement tested. Nevertheless, the commercially available diet supplement tested can undoubtedly serve as a source of natural antioxidants.

### 2.3. Determination of Anti-Hypochlorite Potential

#### 2.3.1. Synthesis of Coumarin-Derived Hypochlorite-Sensitive Fluorescent Probe 7-DCCA

**Figure 3:**
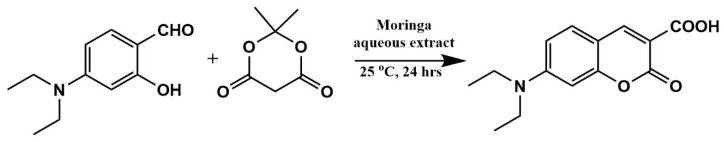
Aqueous Moringa extract-mediated synthesis of the fluorescent probe 7-DCCA.

The anti-hypochlorite potential of aqueous Moringa extract was determined based on the fluorescence quenching of the coumarin-derived hypochlorite-sensitive probe 7-DCCA. This compound was synthesized based on numerous reports, which documented the use of Meldrum’s acid as a substrate for the synthesis of coumarin 3-carboxylic acid derivatives [19,20,21]. A number of these papers refer to various crop-derived products such as juices, extracts, or even waste waters employed as both media and catalysts in the Knoevenagel condensation-like reactions of Meldrum’s acid [5,22]. It is particularly worth emphasizing that the majority of these reactions were carried out at eco-friendly conditions such as room temperature, aqueous media, and only a slightly acidic pH. These requirements were fulfilled by our aqueous Moringa extract and, hence, we attempted a green synthesis of the target compound using the extract as medium and the catalyst in the synthesis of the 7-DCCA probe (Figure 3). The reaction yield was referenced to that of the carried out one in 10% aqueous acetic acid and compared with the previously reported data.

The 7-DCCA product was obtained with a moderate yield of 42%, and was lower than that obtained from the reaction carried out in dilute acetic acid, which resulted in a 56% yield. Additionally, it remained in a stark contrast with excellent yields reported elsewhere [5,22]. A majority of unreacted salicylaldehyde substrate was recovered from the reaction mixture at the recrystallization step, while no unreacted Meldrum’s acid was found. This suggests that the Moringa extract may consists of components with the ability to react with Meldrum’s acid, which lowers the yield of 7-DCCA, even though verification of this hypothesis would require an additional set of experiments. Nevertheless, the room temperature and slight acidic pH of the aqueous Moringa extract were considered sufficient for the isolation of a high purity 7-DCCA product with a satisfactory yield.

#### 2.3.2. Anti-Hypochlorite Assay

Hypochlorite is formed in human neutrophils upon inflammation. Due to its high reactivity, it may damage host cells. Hence, it is important to monitor its presence and remove its excess from cells and body fluids. It is especially important in the case of people suffering from chronic inflammation. Hypochlorous acid (HOCl) belongs to the category of bactericidal and highly reactive chlorine species (RCS). Endogenously, HOCl is formed as a final product on the course of enzymatic reactions occurring in the activated neutrophils phagosomes and is one of the major compounds involved in the activity of the innate immune system [23]. However, its overproduction, e.g., in people diagnosed with chronic inflammation, may lead to numerous and irreversible damage to host cells and tissues. In the acidic interior of the lysosomal vesicles, the HOCl balance moves toward the molecular chlorine Cl_2_ [13]. However, formed promptly and in large quantities, hypochlorous acid can also migrate through the membranes of the phagolysosomal vesicles, and then through the neutrophil membranes to body fluids [24]. The release of residual HOCl and other reactive oxygen species (ROS) from active neutrophils also occurs at the time of their apoptosis after phagocytosis of the pathogen. The pH of body fluids (pH 7.4) shifts the balance of the aqueous HOCl solution toward the formation of hypochlorite ions OCl^−^ [13], while also having strong oxidizing and chlorinating properties. The presence of HOCl and its derivatives in intercellular spaces may lead to the local irritation of epithelial tissue cells, damage of proteins, nucleotides, DNA, RNA, fatty acids, or cholesterol [25,26,27,28]. The harmful effect of hypochlorite on the body is manifested in people suffering from chronic inflammation, e.g., joints, where the immune system is stimulated to produce antibacterial compounds, and, thus, maintains a local concentration of hypochlorite and other ROS on an elevated level, which contributes to further stimulation of the immune system and further increases production of bactericides. It contributes to increasing interest in recent years in the search for compounds capable of detecting an elevated concentration of hypochlorite in tissues and cells *in vivo*, as well as in the search for compounds capable of scavenging it.

Various research reports have identified the ability of coumarin derivatives to penetrate cell membranes [29,30,31,32]. 7-DCCA (Figure 3) is well-known for its fluorescent properties [30,33] and, thus, can be applied as a potential in vivo sensor for biological applications. Our recent studies [34] have shown that the fluorescence intensity of 7-DCCA is quenched linearly with increasing concentration of hypochlorite, which allows it to be potentially used for quantitate determination of hypochlorite at each pH tested. Therefore, to our best knowledge, we have studied, for the first time, the hypochlorite (OCl^−^) ions-scavenging ability of Moringa leaves’ extract using a coumarin-derived probe, 7-(diethylamino) coumarin-3-carboxylic acid (7-DCCA).

**Figure 4:**
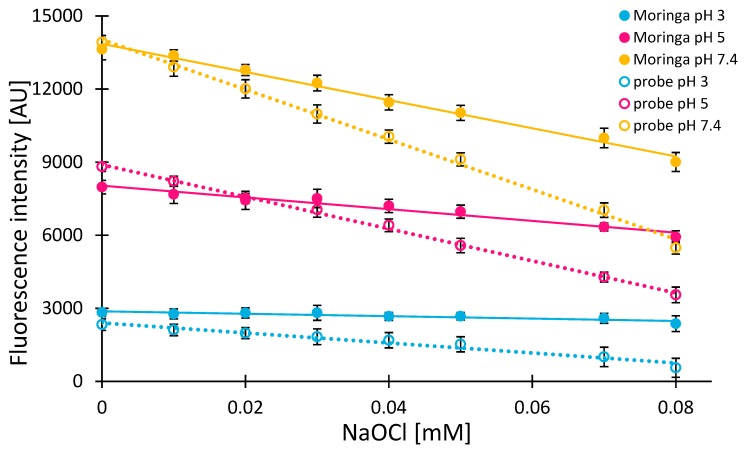
Comparison of changes in fluorescence intensity of 7-DCCA (0.04 mg/mL): under the influence of NaOCl (dotted lines), and, after 15 min of Moringa extract (0.5 mg/mL), incubation with NaOCl (permanent lines). The measurements were carried out at λ_Ex_ 289 nm, λ_Em_ 460 nm, and at a temperature of 25 °C.

The results presented in this study demonstrate the applicability of Moringa extract as an anti-hypochlorite agent and utilize the 7-DCCA probe as a marker of its presence. Various pH conditions occurring in various parts of the human body differentiate the hypochlorous acid forms present in vivo [13]. Therefore, all the experiments were carried out again in three buffers, which are pH’s 3, 5, and 7.4. Figure 4 shows fluorescence intensity changes in 150 μM (0.04 mg/mL) of 7-DCCA solution (dotted lines) upon an increase in the concentration of NaOCl at three pH values.

In each case, a linear decrease in signal intensity was observed, which corresponds well with our previous data. This experiment served as a blank in the determination of anti-hypochlorite activity of the Moringa extract [34]. In the case of the Moringa extract, the samples were tested in an identical manner except that the 7-DCCA probe was added after 15 minutes of extract incubation with NaOCl (Figure 4). In blank samples, the reaction between NaOCl and the probe occurs immediately and remains unchanged once finished (as evidenced in our previous studies) [34]. Therefore, in the case of blank samples, the 15 minute delay was not necessary, while, in the case of Moringa, the 15 minute delay was necessary in order to allow the active compounds from the extract to react with reactive chlorine species.

A direct comparison of results obtained from varied pH samples is difficult due to the fact that the 7-DCCA probe itself shows significant differences in its fluorescence intensity at various environmental conditions. Apparently, the presence of Moringa extract introduces a series of additional intermolecular interactions, which result in varied fluorescence quantum yields of the probe and, thus, differences in the initials fluorescence intensities of 7-DCCA observed [35].

It is clear that the aqueous Moringa extract notably slows down the rate of fluorescence quenching of 7-DCCA at each pH tested after just 15 minutes of the reaction. The slope is given in Figure 4. It reflects the most significant difference in the fluorescence intensity drop, which happens at a pH of 7.4. Therefore, the lowest activity of the extract is at this pH. The slope obtained from measurements carried out at pH 3 points at the highest anti-hypochlorite activity observed in these conditions. This trend may result from a pH-dependent variety of RCS [13], which, in turn, may result in differences in the nature of interactions occurring between RCS and the extract components.

Molecular chlorine, which dominates at a low pH, may react relatively easily with polyphenol-rich extract via an electrophilic substitution mechanism. The chlorination of polyphenols present in the Moringa extract utilizes the Cl_2_ species, which manifests in a persistent fluorescence of the 7-DCCA probe. Compared to Cl_2_, the OCl^−^ species, which dominate at a neutral and basic pH, demonstrate lower reactivity toward phenols. Although the OCl^−^ is well-known for its strong oxidizing character, its concentration seems insufficient for successful oxidation of the polyphenol-rich extract. On the other hand, the OCl^−^ can still chlorinate the 7-DCCA probe [34], which results in quenching of its fluorescence emission. Therefore, the overall anti-hypochlorite activity of the aqueous Moringa extract is high at a low pH, while at neutral and basic conditions, it is relatively lower. These results may vary depending on the solvent used for the extraction from the Moringa powder. Moreover, these finding confirm that the 7-DCCA probe gives a reliable response in a wide pH range.

For better clarity, the results obtained are given as IC_50_ [mM] values (Table 1). The higher the IC_50_ value, the higher the hypochlorite-scavenging activity.

The percentage of 7-DCCA fluorescence quenched is directly proportional to the hypochlorite concentration. Therefore, the higher the concentration of ClO^−^ required to reduce emission intensity of the probe is, the higher the anti-hypochlorite potential of the compound tested is.

The blank samples consisted of only a mixture of NaOCl and the 7-DCCA probe and, regardless of the significant differences in the fluorescence intensity of the probe at various pHs (Figure 4), the amount of NaOCl required to quench 7-DCCA fluorescence by 50% in blank samples was almost identical and oscillated at around 0.07 mM (for 0.15 mM of the 7-DCCA probe in each sample). This corresponds well with our previous studies [34], where equimolar probe: hypochlorite ratio was assumed.

In the case of the Moringa extract-containing samples, the IC_50_ value decreased with an increase in pH, which indicates higher anti-hypochlorite activity at lower pH values. Physiological conditions inside of neutrophil phagocytes in which HOC1 is formed in vivo are characteristic of a pH of 5.0–5.5 [12,13].

It is expected that compounds, which are present in the Moringa extract and particularly those responsible for hypochlorite scavenging, would also demonstrate similar behavior in a living organism. Such a hypothesis is valid based on an assumption that the anti-hypochlorite components of the Moringa extract permeate through the neutrophil cell membranes. Verification of this hypothesis, however, would require a more in-depth research aiming primarily at the identification of those molecules, which react with OCl^−^, and possess the ability to permeate biological membranes [36].

Additionally, promising results were obtained from pH 7.4 samples, where the IC_50_ values were slightly lower compared to those with a pH of 5. The pH of 7.4 reflects the conditions prevailing in body fluids, which are relative easily accessible for any type of active substance from the digestive system. In this context, it is particularly noteworthy that, at a pH of 7.4, only 0.5 mg/mL of aqueous Moringa extract is capable of removing half of the total OCl^−^ amount present in the sample (0.06 mM OCl^−^ required to quench fluorescence in the samples without extract and 0.12 mM OCl^−^ in the sample with extract) (Table 1) after only 15 minutes of incubation.

The results obtained are even more promising once it was noted that all measurements were carried out at 25 °C, which shows the ability of Moringa extract to act at room temperature. It is, therefore, expected that a similar action may take place at a physiological temperature of the human body as well. Increased concentration of extract in the samples together with an extended reaction time with NaOCl in vitro would likely result in further improvement in anti-hypochlorite activity. On the other hand, recent studies state that only 5% to 10% of total phenolic intake can be absorbed from a small intestine [36], which means that effects resulting from the increased extract concentration in vitro may differ from those occurring *in vivo*. Therefore, additional bioavailability studies should be performed in order to determine the maximum concentration of extract necessary for the most effective absorption of its active compounds from the digestive system to the bloodstream.

The anti-hypochlorite activity results obtained from Moringa extract were referenced to those performed with well-known anti-hypochlorite agent ascorbic acid (AA) (see supplementary file Figure S3). The IC_50_ values for 0.005 mg/mL of AA were nearly four to 10 fold higher compared to those of 0.5 mg/mL of Moringa extract (Table 1). However, it is worth noting that AA is highly soluble in water, while Moringa extract is rich in various, active compounds with limited aqueous solubility. Application of organic solvents for extraction significantly improve the final results for the extract, but, again, our goal was estimating the physiological conditions in the experiments performed in vitro.

The available literature consists of countless reports on a variety of biological assays performed on *Moringa oleifera* extracts. On the other hand, the reports on anti-hypochlorite activity of Moringa species are scarce. Thus, the results presented in this study may potentially open a new chapter in researching this plant.

## 3. Materials and Methods

### 3.1. Materials

All chemicals used were of a reagent grade or higher. All solvents were of 99% purity or higher (HPLC grade). DPPH (α,α-diphenyl-β-picrylhydrazyl), Folin-Ciocalteu reagent, TROLOX (6-hydroxy-2,5,7,8-tetramethylchroman-2-carboxylic acid), gallic acid, 4-diethylamino-2-hydroxybenzaldehyde, and Meldrum’s acid were purchased from Sigma Aldrich (St. Louis, MO, USA). Sodium hypochlorite was purchased from ChemPur (Piekary Śląskie, Poland). Methanol and ethanol, ascorbic acid, and sodium carbonate were purchased from Avantor (Gliwice, Poland). *Moringa oleifera* powdered leaves were purchased as commercially available in the European diet supplement Moringa slim (Azurelab Holdings Ltd., Nicosia, Cyprus).

### 3.2. Instrumentation

Steady-state fluorescence measurements and UV-Vis spectrophotometric measurements were performed on a Tecan Infinite 200 microplate reader (Tecan Austria GmbH, Grödig/Salzburg, Austria). The NMR spectrum of the coumarin-derived probe was acquired on a Bruker Avance III spectrometer (500 MHz), using DMSO^−^d6 as a solvent. The infrared spectra were recorded in the region of 4000 cm^−1^ to 400 cm^−1^ on a Shimadzu IR Spirit Fourier-Transform Infrared spectrophotometer equipped with an ATR adapter. HPLC-ESI-MS analyses were performed on an LCMS-8030 mass spectrometer (Shimadzu, Kyoto, Japan), according to the method described previously [34]. All HPLC-MS analyses were performed in a positive ion mode.

### 3.3. Methods

#### 3.3.1. Extract Preparation

The human body is primarily an aquatic environment. Therefore, in order to imitate the physiological conditions during all experiments performed, all Moringa extracts were prepared in water without any organic solvents. Additionally, 100 mg of powdered leaves were suspended in 10 mL of demineralized water. The mixture was then vigorously shaken and kept for extraction in room temperature for 15 min, and then centrifuged at 5000 rpm for 10 min. The supernatant was collected and stored at −18 °C for total phenolic content determination, antioxidant, and anti-hypochlorite assays.

#### 3.3.2. Total Phenolic Content Determination

Total phenolic content (TPC) determination of aqueous Moringa extract was performed using a modified Folin-Ciocalteu assay. A volume of 0.5 mL of Moringa extract (5 mg/mL) was mixed with 0.5 mL of Folin-Ciocalteu reagent (FCR). After 3 min at 25 °C, 1.5 mL of sodium carbonate solution (saturated) was added and the sample was filled with water up to 50 mL. The absorbance was measured at λ_max_ 735 nm after 30 min of incubation of samples in a water bath at 40 °C. The results were presented in grams of gallic acid equivalents (GAE) per 100 g of the sample dry weight.

#### 3.3.3. Antioxidant Assay

To the following wells of transparent, 96-well plate, increasing concentrations of Moringa solution was applied. Therefore, its final concentration in 200 μL of the total volume of each sample ranged from 0 to 0.5 mg/mL. Samples of the reference compound, which were ascorbic acid (AA) and TROLOX (TROL), were prepared the same way and their final concentration in 200 μL of the total volume of each sample ranged from 0 to 0.009 mg/mL and 0 to 0.015 mg/mL, respectively. Just before the measurement, all wells were supplemented with 1 mM ethanolic solution of DPPH^•^ radicals so that their final concentration in each well was 200 μM. The plate was shaken on an internal shaker of the reader for 10 s to ensure thorough mixing of reagents in all wells. The spectrophotometric measurements started in the range of λ 500–550 nm with a 1 nm wavelength step at 25 °C for 30 min. The results obtained were the average of five exposures of each sample with a beam of light. All experiments were repeated three times.

#### 3.3.4. Synthesis of 7-Diethylamino-Coumarin-3-Carboxylic Acid

The suspension of 1g Moringa powder in 100 mL of demineralized water was subjected to ultrasound irradiation at room temperature for 10 min and the solid was then filtered off. The filtrate was used as a medium and catalyst for the synthesis of 7-diethylamino-coumarin-3-carboxylic acid.

4-diethylamino-2-hydroxybenzaldehyde (1.0 g, 5 mmol) and Meldrum’s acid (0.75 g, 5 mmol) was dissolved in 30 mL of aqueous Moringa extract and stirred at room temperature for 24 h. The orange solid formed was filtered off, dried, and recrystallized from methanol, which yielded dark-orange crystals of 7-diethylamino-coumarin-3-carboxylic acid: Yield 0.56 g (42%), C_14_H_15_NO_4_ (261.28 g/mol), *m/z* [M+H]^+^ 262.00, ^1^H-NMR (DMSO): δ = 12.48 ppm (s, 1H, H3 (-COOH)), 8.58 (s, 1H, H4), 7.64 (d, 1H, H5, *J* = 9.04 Hz), 6.80 (dd, 1H, H6, *J_1_* = 9.04 Hz, *J_2_* = 2.30 Hz), 6.57 (d, 1H, H8 *J* = 2.30 Hz), 3.49 (q, 4H, (-CH_2_-)), 1.14 (t, 6H, (-CH_3_)).

#### 3.3.5. Anti-Hypochlorite Assay

To the fallowing wells of black, 96-well plate, 20 μL of 25 mM acetate (pH 3 and 5) or phosphate (pH 7.4) buffers and NaOCl were added. The final concertation of hypochlorite in the total volume (200 μL) of samples ranged from 0 to 0.84 mM. Next, 10 μL of Moringa extract solution was applied and, after 15 min of a reaction, 30 μL of 1 mM probe solution was added. The plate was shaken for 10 s on the reader shaker and then the fluorescence measurement was started. All samples were excited by light at a wavelength of λ_Exc_ 289 nm and the fluorescence spectra at λ 320–700 nm were recorded at 25 °C. The results obtained were the average of five exposures of each sample with a beam of light. All experiments were repeated three times. Analysis for the reference substance ascorbic acid 10 μL of 0.1 mg/mL solution in each well were made analogously.

## 4. Conclusions

Total phenolic content determined in the *Moringa oleifera* sample tested was 4.04 ± 0.11 g GAE/100 g DW. The antioxidative potential determined for the aqueous extract is notably lower than that of the standard (TROLOX), which may result from the low aqueous solubility of compounds responsible for radical scavenging.

The research carried out for the first time to determine the ability of Moringa leaves extract to scavenge hypochlorous ions in vitro gave satisfactory results. At the pH value adequate to that of the body fluid environment (7.4), the extract was able to reduce the OCl^−^ ions concentration present in the solution by 50% after 15 min of the reaction carried out at 25 °C. Considering the preliminary stage of research carried out, this is undoubtedly a satisfactory result. Understanding the mode of action of the extract, however, requires a more elaborate approach.

The research reported in this study aimed at examining a commercially available *Moringa oleifera* dietary supplement for its ability to provide an effect similar to that attributed to fresh plant extracts. The results obtained allow the assumption that the benefits for human health properties of *Moringa oleifera* species can also be enjoyed by people living in areas where this plant does not occur endemically.

## Figures and Tables

**Table 1:** IC_50_ value of NaOCl concentration required to quench fluorescence of 150 μM of 7-DCCA by 50% with or without the presence of compounds with potential anti-hypochlorite activity. Results obtained after 15 minutes of analytes incubation with an increasing concentration of NaOCl at 25 °C.

		IC_50_ [mM]	
	pH 3	pH 5	pH 7.4
Moringa _(aq)_	0.41 ± 0.02	0.18 ± 0.01	0.12 ± 0.01
7-DCCA ^1^	0.07 ± 0.00	0.07 ± 0.00	0.06 ± 0.00
ascorbic acid ^2^	1.81 ± 0.09	1.11 ± 0.07	1.22 ± 0.13

^1^ blank, ^2^ reference.

## Supplementary Materials

The following are available online. Figure S1: DPPH reduction percentage of ascorbic acid at various pH values after 30 minutes of reaction at 25 °C. Figure S2: DPPH reduction percentage of TROLOX at various pH values after 30 min of reaction at 25 °C. Figure S3: Changes in fluorescence intensity of 7-DCCA (0.04 mg/mL) after 15 min of ascorbic acid (0.005 mg/mL) incubation with NaOCl. The measurements were carried out at λ_Ex_ 289 nm, λ_Em_ 460 nm, and at a temperature of 25 °C.

## Abbreviations

7-DCCA	7-diethylamino-coumarin-3-carboxylic acid
AA	ascorbic acid
DW	dry weight
FCR	Folin–Ciocalteu reagent
GAE	gallic acid equivalents
OCl^−^	hypochlorite ion
RCS	reactive chlorine species
ROS	reactive oxygen species
TPC	total phenolic content
TROL	TROLOX

## References

[1] Gopalakrishnan L., Doriya K., Kumar D.S. (2016). *Moringa oleifera*: A review on nutritive importance and its medicinal application. Food Sci. Hum. Wellness.

[2] Leone A., Spada A., Battezzati A., Schiraldi A., Aristil J., Bertoli S. (2015). Cultivation, Genetic, Ethnopharmacology, Phytochemistry and Pharmacology of *Moringa oleifera* Leaves: An Overview. Int. J. Mol. Sci..

[3] Popoola J.O., Obembe O.O. (2013). Local knowledge, use pattern and geographical distribution of *Moringa oleifera* Lam. (Moringaceae) in Nigeria. J. Ethnopharmacol..

[4] Anwar F., Lafit S., Ashraf M., Gilani A.H. (2007). *Moringa oleifera*: A Food Plant with Multiple Medicinal Uses. Phytother. Res..

[5] Bagul S.D., Rajput J.D., Bendre R.S. (2017). Synthesis of 3-carboxycoumarins at room temperature in water extract of banana peels. Environ. Chem. Lett..

[6] Iqbal S., Bhanger M.I. (2006). Effect of season and production location on antioxidant activity of *Moringa oleifera* leaves grown in Pakistan. J. Food Compos. Anal..

[7] Pakade V., Cukrowska E., Chimuka L. (2013). Comparison of antioxidant activity of *Moringa oleifera* and selected vegetables in South Africa. S. Afr. J. Sci..

[8] Siddhuraju P., Becker K. (2003). Antioxidant Properties of Various Solvent Extracts of Total Phenolic Constituents from Three Different Agroclimatic Origins of Drumstick Tree (*Moringa oleifera* Lam.) Leaves. J. Agric. Food Chem..

[9] Vergera-Jimenez M., Almatrafi M.M., Fernandez M.L. (2017). Bioactive Components in *Moringa Oleifera* Leaves Protect against Chronic Disease. Antioxidants.

[10] Cieślik E., Gręda W., Adamus W. (2006). Contents of polyphenols in fruit and vegetables. Food Chem..

[11] Pérez-Jiménez J., Neveu V., Vos F., Scalbert A. (2010). Identification of the 100 richest dietary sources of polyphenols: An application of the Phenol-Explorer database. Eur. J. Clin. Nutr..

[12] Klebanoff S.J. (2005). Myeloperoxidase: Friend and foe. J. Leukoc. Biol..

[13] Kettle A.J., Albrett A.M., Chapman A.L., Dickerhof N., Forbes L.V., Khalilova I., Turnet R. (2014). Measuring chlorine bleach in biology and medicine. Biochim. Biophys. Acta.

[14] Pękal A., Pyrzynska K. (2015). Effect of pH and metal ions on DPPH radical scavenging activity of tea. Int. J. Food Sci. Nutr..

[15] Dawidowicz A.L., Olszowy M. (2012). Mechanism change in estimating of antioxidant activity of phenolic compounds. Talanta.

[16] Michiels J.A., Kevers C., Pincemail J., Defraigne J.O., Dommes J. (2012). Extraction conditions can greatly influence antioxidant capacity assays in plant food matrices. Food Chem..

[17] Wright R.J., Lee K.S., Hyacinth H.I., Hibbert J.M., Reid M.E., Wheatley A.O., Asemota H.N. (2017). An Investigation of the Antioxidant Capacity in Extracts from *Moringa oleifera* Plants Grown in Jamaica. Plants.

[18] Sreelatha S., Padma P.R. (2009). Antioxidant Activity and Total Phenolic Content of Moringa oleifera Leaves in Two Stages of Maturity. Plant Foods Hum. Nutr..

[19] Song A., Wang X., Lam K.S. (2003). A convenient synthesis of coumarin-3-carboxylic acids via Knoevenagel condensation of Meldrum’s acid with *ortho*-hydroxyaryl aldehydes or ketones. Tetrahedron. Lett..

[20] Ghalehshahi H.G., Balalaie S., Aliahmadi A. (2018). Peptides N-Connected to Hydroxycoumarin and cinnamic acid derivatives: Synthesis and Fluorescence Spectroscopic, Antioxidant and Antimicrobial Properties. New J. Chem..

[21] Brahmachari G. (2015). Room Temperature One-Pot Green Synthesis of Coumarin-3-carboxylic Acids in Water: A Practical Method for the Large-Scale Synthesis. ACS Sustain. Chem. Eng..

[22] Fiorito S., Taddeo V.A., Genovese S., Epifano F. (2016). A green chemical synthesis of coumarin-3-carboxylic and cinnamic acids using crop-derived products and waste waters as solvents. Tetrahedron. Lett..

[23] Zhang R., Song B., Youan J. (2018). Bioanalytical methods for hypochlorous acid detection: Recent advances and challenges. Trends Anal. Chem..

[24] Hampton M.B., Kettle A.J., Winterbourn C.C. (1998). Inside the Neutrophil Phagosome: Oxidants, Myeloperoxidase, and Bacterial Killing. Blood.

[25] Albrich J.M., McCarthy C.A., Hurst J.K. (1981). Biological reactivity of hypochlorous acid: Implications for microbicidal mechanisms of leukocyte myeloperoxidase. Proc. Natl. Acad. Sci. USA.

[26] Dennis W.H., Olivieri V.O., Krusé C.W. (1979). The reaction of nucleotides with aqueous hypochlorous acid. Water Res..

[27] Visser M.C.M., Winterbourn C.C. (1991). Oxidative damage to fibronectin: I. The effects of the neutrophil myeloperoxidase system and HOCl. Arch. Biochem. Biophys..

[28] Carr A.C., Vissers M.C.M., Domigan N.M., Winterbourn C.C. (1997). Modification of red cell membrane lipids by hypochlorous acid and haemolysis by preformed lipid chlorohydrins. Redox Rep..

[29] Wang L., Li W., Zhi W., Ye D., Zhang W., Ni L. (2018). Rapid detection of hypochlorite by a coumarin-based hydrazide in aqueous solution and its application in live-cell imaging. Sens. Actuators B Chem..

[30] Long L., Wu Y., Wang L., Gong A., Hu F., Zhang C. (2015). A Fluorescent Probe for Hypochlorite Based on Modulating the Unique Rotation of N-N Single Bond in Acetohydrazide. Chem. Commun..

[31] Liao Y.C., Venkatesan P., Wei L.F., Wu S.P. (2016). A coumarin-based fluorescent probe for thiols and its application in cell imaging. Sens. Actuators B Chem..

[32] Yu S.Y., Hsu C.Y., Chen W.C., Wei L.F., Wu S.P. (2014). A hypochlorous acid turn-on fluorescent probe based on HOCl-promoted oxime oxidation and its application in cell imaging. Sens. Actuators B Chem..

[33] Chatterjee A., Seth D. (2013). Photophysical Properties of 7-(diethylamino)Coumarin-3-carboxylic Acid in the Nanocage of Cyclodextrins and in Different Solvents and Solvent Mixtures. Photochem. Photobiol..

[34] Starzak K., Matwijczuk A., Creaven B., Matwijczuk A., Wybraniec S., Karcz D. (2019). Fluorescence Quenching-Based Mechanism for Determination of Hypochlorite by Coumarin-Derived Sensors. Int. Mol. J. Sci..

[35] Brewer W.D., Haken H., Wolf H.C. (2013). Molecular Physics and Elements of Quantum Chemistry: Introduction to Experiments and Theory.

[36] Chen L., Cao H., Xiao J., Galanakis C.M. (2018). 2-Polyphenols: Absorption, bioavailability, and metabolomics. Polyphenols: Properties, Recovery, and Applications.

