# Supplementary material for: Anti-Hypochlorite and Catalytic Activity of Commercially Available Moringa oleifera Diet Supplement
Source: Molecules. 2019 Sep 12;24(18):3330. doi: 10.3390/molecules24183330 (PMC6767131; doi:10.3390/molecules24183330)
Supplement: Supplementary file 1 [file molecules-24-03330-s001.pdf]

Supplementary materials:

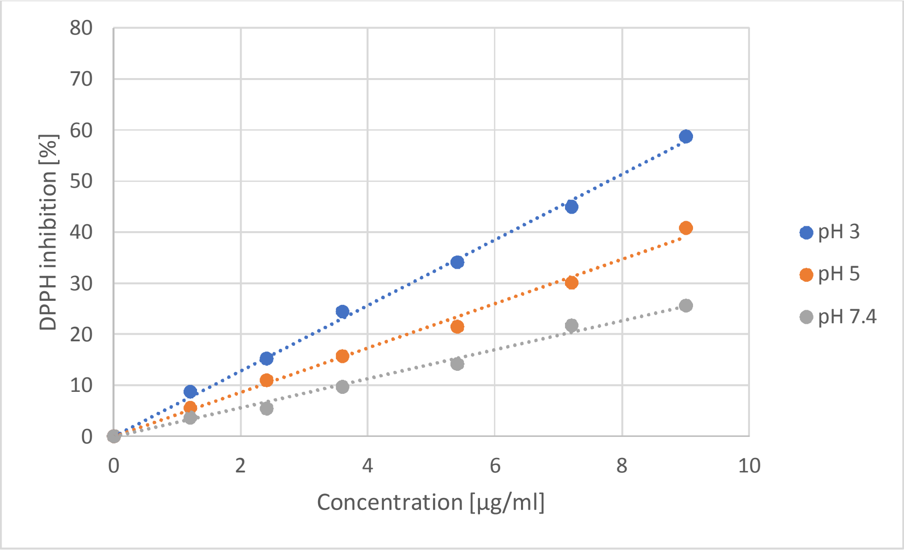

Figure S1. DPPH reduction percentage of ascorbic acid at various pH values after 30 min of reaction at 25 ^oC.

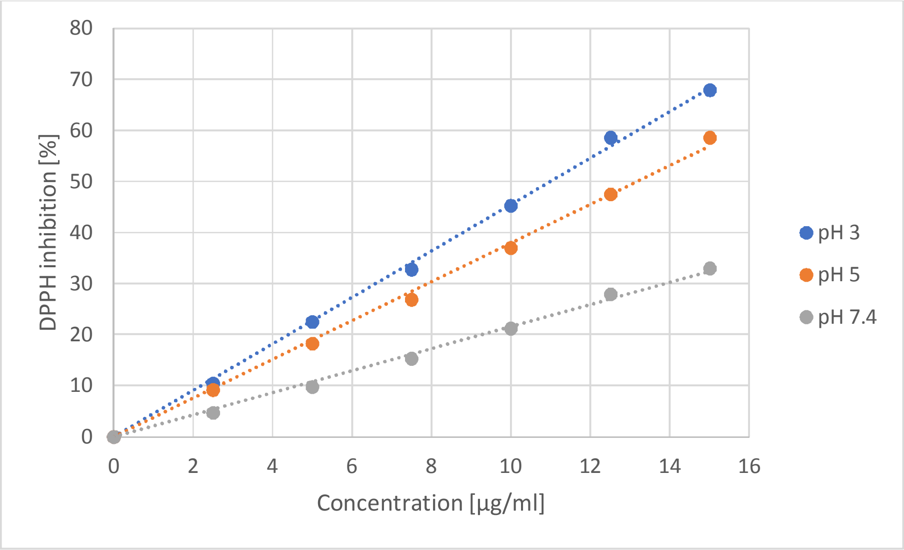

Figure S2. DPPH reduction percentage of Trolox at various pH values after 30 min of reaction at 25 ^oC.

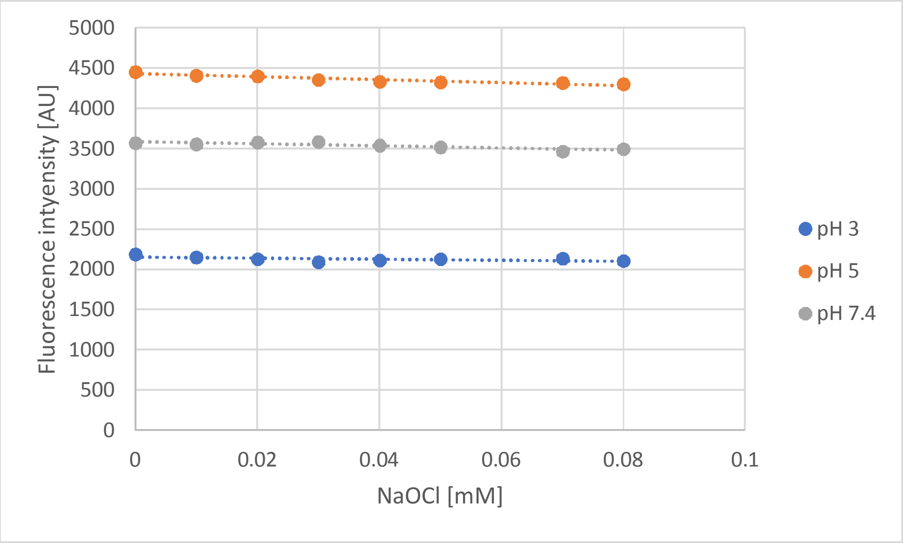

Figure S3. Changes in fluorescence intensity of 7-DCCA (0.04 mg/ml) after 15 minutes of ascorbic acid (0.005 mg/ml) incubation with NaOCl. The measurements were carried out at λ_{Ex} 289 nm, λ_{Em} 460 nm, and at a temperature of 25 °C.
